# Biogeochemical dynamics and microbial community development under sulfate- and iron-reducing conditions based on electron shuttle amendment

**DOI:** 10.1371/journal.pone.0251883

**Published:** 2021-05-20

**Authors:** Theodore M. Flynn, Dionysios A. Antonopoulos, Kelly A. Skinner, Jennifer M. Brulc, Eric Johnston, Maxim I. Boyanov, Man Jae Kwon, Kenneth M. Kemner, Edward J. O’Loughlin

**Affiliations:** 1 Biosciences Division, Argonne National Laboratory, Lemont, Illinois, United States of America; 2 Institute of Chemical Engineering, Bulgarian Academy of Sciences, Sofia, Bulgaria; 3 Department of Earth and Environmental Sciences, Korea University, Seoul, South Korea; The University of Akron, UNITED STATES

## Abstract

Iron reduction and sulfate reduction are two of the major biogeochemical processes that occur in anoxic sediments. Microbes that catalyze these reactions are therefore some of the most abundant organisms in the subsurface, and some of the most important. Due to the variety of mechanisms that microbes employ to derive energy from these reactions, including the use of soluble electron shuttles, the dynamics between iron- and sulfate-reducing populations under changing biogeochemical conditions still elude complete characterization. Here, we amended experimental bioreactors comprised of freshwater aquifer sediment with ferric iron, sulfate, acetate, and the model electron shuttle AQDS (9,10-anthraquinone-2,6-disulfonate) and monitored both the changing redox conditions as well as changes in the microbial community over time. The addition of the electron shuttle AQDS did increase the initial rate of Fe^III^ reduction; however, it had little effect on the composition of the microbial community. Our results show that in both AQDS- and AQDS+ systems there was an initial dominance of organisms classified as *Geobacter* (a genus of dissimilatory Fe^III^-reducing bacteria), after which sequences classified as *Desulfosporosinus* (a genus of dissimilatory sulfate-reducing bacteria) came to dominate both experimental systems. Furthermore, most of the ferric iron reduction occurred under this later, ostensibly “sulfate-reducing” phase of the experiment. This calls into question the usefulness of classifying subsurface sediments by the dominant microbial process alone because of their interrelated biogeochemical consequences. To better inform models of microbially-catalyzed subsurface processes, such interactions must be more thoroughly understood under a broad range of conditions.

## Introduction

The biogeochemical cycling of carbon (C), iron (Fe), and sulfur (S) in aquatic and terrestrial environments is driven largely by microbially-catalyzed redox reactions. Such reactions by definition involve the transfer of electrons, so it is necessary to assess the thermodynamic and kinetic constraints on electron transfer in appropriate model systems in order to understand the metabolic processes that drive these biogeochemical cycles. For example, in many environments ferric iron (Fe^III^) is primarily present as relatively insoluble Fe^III^ oxides. These minerals provide an important electron sink during anaerobic respiration by a variety of dissimilatory Fe^III^-reducing bacteria (DIRB) and archaea. These phylogenetically diverse microorganisms are able to obtain energy by coupling the oxidation of organic compounds or molecular hydrogen to the reduction of Fe^III^ to Fe^II^ under suboxic and anoxic conditions [1–3]. Fe^III^-reducing microorganisms and the reactive ferrous species they produce play a major role in controlling water quality [4,5], the dissolution and precipitation of minerals [6–8], nutrient availability [9], and the fate and transport of contaminants [10].

Due to the relative insolubility of ferric minerals in most environments, DIRB must employ different mechanisms to respire using these terminal electron acceptors than those used for soluble terminal electron acceptors such as dissolved oxygen (O_2_), nitrate (NO_3_^−^), and sulfate (SO_4_^2−^) [11]. Some DIRB such as *Geobacter* and *Shewanella* can transfer electrons directly to Fe^III^ oxide surfaces by means of reductases located on their outer cell membrane [12] or via electrically conductive pili or nanowires [13–17]. The need for physical contact between Fe^III^ oxide minerals and microbial cells, however, can be readily overcome. The dissolution of Fe^III^ oxides is promoted by exogenous and endogenous ligands and the resulting soluble Fe^III^ complexes can diffuse away and be reduced by DIRB at a distance [18,19]. Likewise, the transfer of electrons from the cell to external electron acceptors (e.g., Fe^III^ oxides) can be facilitated by soluble electron shuttles, i.e., compounds that can be reversibly oxidized and reduced. In this scenario, an oxidized electron shuttle is reduced by the organism, which can transfer electrons to a remote acceptor. Since electron shuttles can be oxidized and reduced repeatedly, they can have a substantial effect on both the rate and extent of Fe^III^ oxide reduction even when present at trace concentrations [20].

A wide variety of endogenous and exogenous organic and inorganic compounds have been shown to function as electron shuttles in the bioreduction of Fe^III^ oxides, including quinones, flavins, phenazines, and reduced sulfur species [20–31]. In addition, humic substances—a class of naturally occurring, chemically heterogeneous organic oligoelectrolytes derived primarily from the decomposition of bacteria, algae, and higher plant material that are ubiquitous in aquatic and terrestrial environments—can also be utilized as electron shuttles in the bioreduction of Fe^III^ oxides [18,30,32–35]. The ability of humic substances to act as electron shuttles has largely been attributed to the presence of quinone groups within their structures [36–38]. However, given the polymorphic nature of humic substances, their structure and functional characteristics are highly variable, including the type and number of reversibly redox active moieties, resulting in a distribution of redox potentials and inherent variability in the redox properties observed among them [38–42]. Therefore, model quinones with well-defined redox characteristics (e.g., reduction potentials) such as 9,10-anthraquinone-2,6-disulfonate (AQDS) have been widely used as analogs for the redox active moieties in humic substances [6,32,43].

A phylogenetically diverse range of bacteria and archaea can transfer electrons to model quinones (e.g., AQDS) and humic substances [44,45]. Indeed, many microorganisms that are not able to reduce Fe^III^ oxides directly, can cause the reduction of Fe^III^ oxides in the presence of a suitable electron shuttle, including organisms that are not primarily categorized as Fe^III^-reducing microbes (i.e., fermenters, sulfate reducers, and methanogens) [26,27,44,46–49]. The ubiquity of humic/quinone-reducing microorganisms in aquatic and terrestrial environments [50] and the ability of reduced humics/quinone to shuttle electrons to Fe^III^ oxides suggests that the presence of electron shuttles provides the potential for bacteria that are not metabolically capable of reducing Fe^III^ minerals to contribute to Fe^III^ oxide bioreduction, thereby increasing microbial diversity under iron-reducing conditions. Although electron shuttles have been shown to significantly enhance Fe^III^ oxide reduction in systems with complex, multispecies microbial communities [51–55], the effects of electron shuttles on microbial community development have not been explicitly examined. In this study, we investigate the effects of the presence and absence of a soluble electron shuttle (AQDS) on biogeochemical dynamics and microbial community development under Fe^III^- and sulfate-reducing conditions.

## Materials and methods

### Experimental setup

Experimental bioreactors were created in triplicate using 500 mL serum bottles containing 400 mL of sterile defined mineral medium (pH 7.5). This medium was comprised of HEPES buffer (20 mM), PIPES buffer (20 mM), sodium acetate (10 mM), Na_2_SO_4_ (5 mM), CaCl_2_ (5 mM), MgCl_2_ (1 mM), KCl (0.5 mM), NH_4_Cl (1 mM), Na_2_HPO_4_ (10 μM), NaHCO_3_ (30 mM), and 10 mL L^–1^ of trace minerals solution [20]. Fe^III^ was provided as natural sienna (Earth Pigments Co.), an iron-rich earth mined from ochre deposits in the Provence region of France that consists primarily of quartz and goethite (α-FeOOH), as determined by powder x-ray diffraction (pXRD) and Fe K-edge extended x-ray absorption fine-structure (EXAFS) spectroscopy. Details of the characterization of natural sienna are provided in Supporting Information (S1 File). Natural sienna was added to the medium at a concentration of 7.6 g L^–1^; which is equivalent to a total of 30 mmol Fe^III^ per liter of medium. Sieved (400 mesh) quartz (SiO_2_) was also added at a concentration of 72 g L^–1^ (Alfa Aesar). In electron shuttle-amended bioreactors, AQDS was added to a final concentration of 100 μM from a sterile, anaerobic stock solution.

Serum bottles were sparged with sterile, O_2_-free argon gas and inoculated with 10 grams of sediment obtained from a depth of 7 meters below land surface in a shallow, unconfined alluvial aquifer at the Integrated Field Research Challenge (IFRC) site located at the Old Rifle site (39°31’44.864"N 107°46’20.154"W), which is located approximately 0.3 miles east of the city of Rifle in western Colorado [56]. The IFRC is owned by the City of Rifle and The US Department of Energy Office of Legacy Management and its associated researchers were provided access to the site by the City of Rifle through a letter of agreement” [56]. Sediment was taken from sampling well LR-MLS-21 [57] and transported to the lab under anoxic conditions, where it was kept refrigerated at 4 °C until added to the bioreactors. Once inoculated, the bioreactors were sealed with plug septa and aluminum crimp caps. The bottles were secured in flask clamps mounted on a roller drum (Bellco Glass, Inc.) and rotated vertically as the long axis of the bottle remained in a horizontal orientation and were incubated in the dark at 25 °C. Sterilized (autoclaved, 1 cycle) control bottles were used to test for reduction in the absence of microbial activity.

Experimental systems were subsampled periodically using sterile syringes to measure the production of Fe^II^, the consumption of acetate and sulfate, and changes in pH. Additional samples were taken at the same time to measure total protein as well as to extract DNA for 16S rRNA gene amplicon sequencing. Unless otherwise indicated, sample collection and processing were conducted in an anoxic glove box (Coy Laboratory Products) containing an anoxic atmosphere (N_2_:H_2_ = 95:5, O_2_ < 1 ppm) and treated as described below for specific geochemical and microbiological assays. Subsamples for microbial community analysis were frozen at −80 °C and stored at this temperature until DNA was extracted.

### Geochemical analyses

The reduction of Fe^III^ in the bioreactors was monitored by measuring the production of Fe^II^ over time. Samples for total Fe^II^ (i.e., dissolved Fe^II^ and acid-extractable Fe^II^) analysis were prepared by adding 0.75 mL of anoxic 1 M HCl to a 0.25 mL subsample of the well-mixed bioreactor suspension. The samples were mixed on an end-over-end shaker for two weeks, and then centrifuged at 25,000 × *g* for 10 min. The concentration of Fe^II^ in the supernatant was determined spectrophotometrically using the ferrozine assay [58]. Briefly, 1 mL of HEPES-buffered ferrozine reagent [59] was added to 50 μL of supernatant and the absorbance was measured at 562 nm using a Cary 100 UV-Vis spectrophotometer. The detection limit for ferrous iron using this method is approximately 10 μM.

Samples for sulfate and acetate analysis were prepared by centrifuging a subsample of the well-mixed suspension at 25,000 × *g* for 10 min, then removing 50 μL of supernatant and combining it with 950 μL of an isopropanol solution (15% v:v) to preserve the sample until analysis. The concentrations of sulfate and acetate were measured using a Dionex ICS 3000 ion chromatograph equipped with an IonPac AS11 analytical column (250 × 2 mm, Dionex) and a 1-20 mM KOH eluent gradient at a flow rate of 0.5 mL min^−1^. The detection limit for these anions was approximately 1 μM.

### X-ray absorption spectroscopy

Fe K-edge (7,112 eV) x-ray absorption spectroscopy measurements were carried out at the MR-CAT/EnviroCAT bending magnet beamline (Sector 10, Advanced Photon Source) [60]. The reactor solids were separated by filtration through a 0.22 μm PTFE filter inside the anoxic glove box and the hydrated filter cake was sealed together with the filter between two layers of Kapton film; the filtrate was saved for measurement of dissolved FeII using the ferrozine assay. X-ray absorption near-edge spectra (XANES) and extended x-ray absorption fine structure (EXAFS) spectra were collected at room temperature from the standards and the reactor solids inside a N_2_-purged sample cell [61,62]. Anoxic integrity of samples prepared and measured this way have been demonstrated in previous work [63]. Energy calibration was established by setting the inflection point in the spectrum from an Fe foil to 7,112 eV and maintained continuously afterwards by collecting data from the foil simultaneously with the collection of data from the samples. Radiation-induced changes in the spectra were not observed. No differences were observed between spectra from different areas on the sample so all scans from each sample were averaged to produce the final spectrum. Analysis of the spectra involved comparisons to standards followed by linear combination (LC) fitting to extract the spectral weight of up to three standards that best describe the experimental spectra of the bioreactors. More details on the standards and analysis are included in the Supporting Information (S1 File).

### DNA extraction, amplification, and sequencing

The samples for microbial community analysis were kept frozen at −80 °C until use, when they were thawed for 10 min in a 70 °C water bath, then centrifuged at 3,716 × *g* for 10 min. The supernatant was removed, and DNA was extracted from the remaining sediment (~1 g) using the Power Soil DNA Isolation Kit (QIAGEN). Bacterial DNA amount for each sample was then normalized for the overall biomass value at each sample time point. Multiple displacement amplification was performed utilizing phi29 with the GenomiPhi V2 DNA Amplification Kit (GE Healthcare). Protein amounts were also determined for each sample using the bicinchoninic acid-copper (BCA) assay [64] to be used as a marker of biomass value.

Amplicon libraries spanning the V3-V4 region of the 16S rRNA encoding gene (338–802) were constructed using primers to target members of the domain *Bacteria*. The primers spanned *Escherichia coli* positions 338–802 using 338F (5’-ACTCCTACGGGAGGCAGC-3’) and equimolar amounts of the 802R reverse primers (802R-A 5’-TACCRGGGTHTCTAATCC-3’, 802R-B 5’-TACCAGAGTATCTAATTC-3’, 802R-C 5’-CTACDSRGGTMTCTAATC-3’, 802R-D 5’-TACNVGGGTATCTAATCC-3’) from the Ribosomal Database Project’s (RDP’s) Pipeline [65]. Permuted primers containing 10-bp sequences between the sequence adapter (454 Life Sciences A) and the 16S rRNA primer sequence on the forward primer were used to sequence multiple libraries within the same run. PCR reactions were carried out in triplicate for each sample using Platinum^®^ *Taq* High Fidelity polymerase (Invitrogen, ThermoFisher). PCR conditions used an initial denaturation step of 95 °C for 3 min, followed by 30 cycles of 95 °C for 30 s, 57 °C for 45 s, then 72 °C for 1 min and finalized by a single extension step 72 °C for 2 min. Pooled triplicate product for each sample was then purified using the QIAGEN MinElute^®^ PCR Purification Kit. Amplicon presence, sizing, and concentration was assessed using the Agilent Bioanalyzer DNA 1000 BioChip and equimolar sample pools were prepared for sequencing. 16S rRNA gene amplicons were then sequenced using the 454 Life Sciences Genome Sequencer FLX System and following the manufacturer’s protocols. All sequencing and data generation was performed by 454 Life Sciences (Roche) utilizing the XLR70 (Titanium) sequencing chemistry.

Sequencing of 16S rRNA gene amplicons produced a total of 243,565 sequences across 38 samples. Following sequencing, amplicon libraries were processed using a combination of QIIME [66], Acacia [67], and UPARSE [68]. Sequences were demultiplexed and quality filtered using QIIME version 1.9.1. Sequences of poor quality were discarded based on divergence from expected amplicon length (470 bp), quality scores (minimum score 25), long homopolymer runs (max = 6), and primer mismatches (max = 0). All sequences not meeting quality standards (26,305, or 10.7% of the original total) were discarded. Remaining libraries maintained an average sequencing depth of 5,717 ± 2,304 reads.

Remaining sequences were denoised using Acacia then screened for chimeric sequences, dereplicated, and clustered into operational taxonomic units (OTUs) at a 97% similarity cutoff in UPARSE using the -cluster_otus command and the UPARSE-OTU algorithm. Taxonomic identities were assigned to representative sequences from each OTU using the SILVA reference database (version 128) [69]. Singleton OTUs (those containing only a single sequence across all samples) were discarded prior to downstream analyses. Alpha and beta diversity analyses were conducted in the R programming environment using the packages phyloseq [70] and edgeR [71] as well as Primer-7 [72]. Raw sequence data are publicly available through MG-RAST [73] under project number mgp96975.

## Results

### Geochemical changes in sediment bioreactors

**Fig 1:**
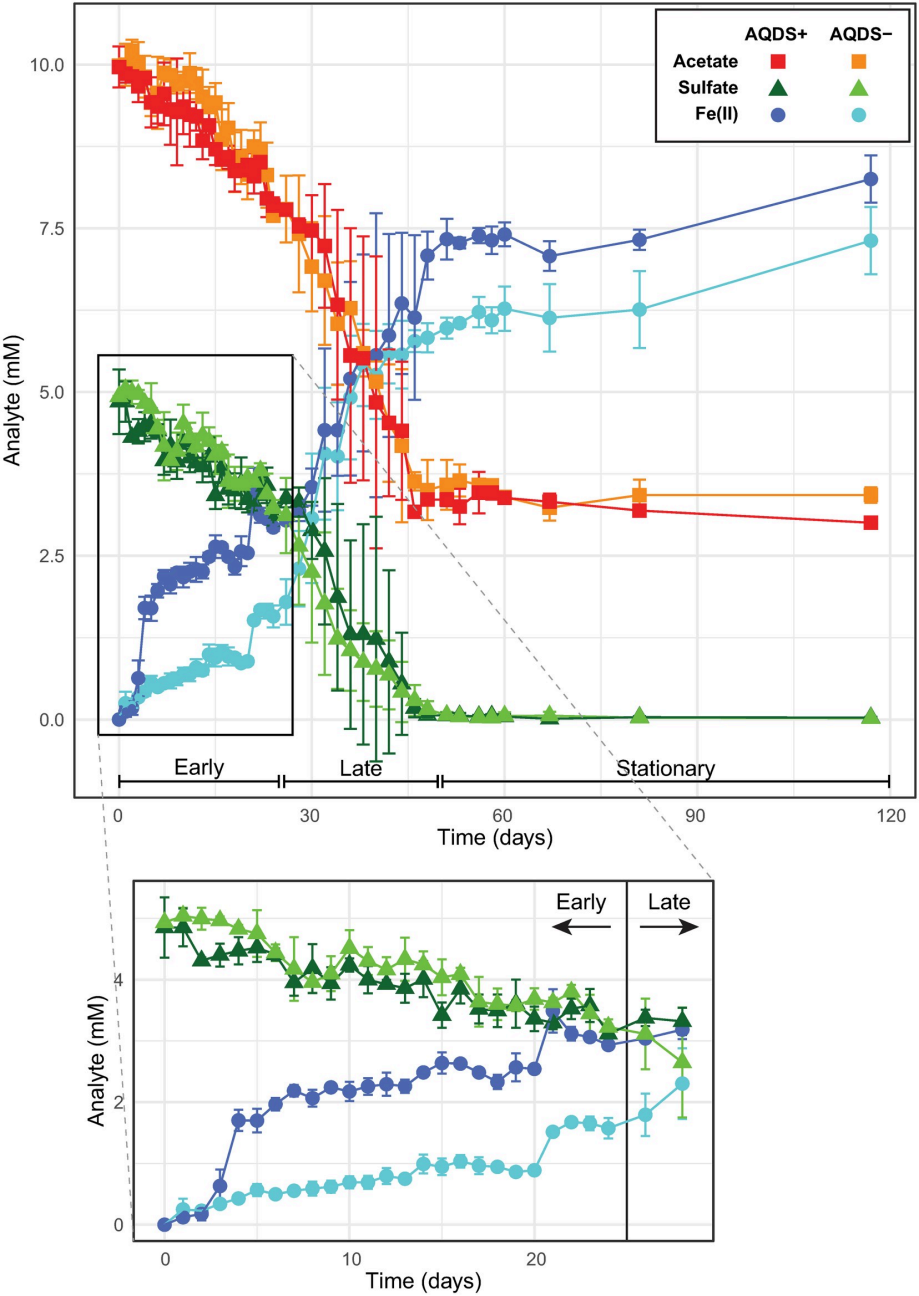
Analyte concentrations in bioreactors. Concentrations of total ferrous iron (Fe^II^), acetate, and sulfate over time in AQDS–amended (AQDS+) and control bioreactors (AQDS–) with an expanded view of Fe^II^ and sulfate concentrations from 0–28 days.

Bioreactors amended with AQDS were compared to unamended controls, monitoring the levels of acetate, Fe^II^, and sulfate over time. The overall trajectory of these changes can be seen in Fig 1, which can be separated into three distinct phases: an “early” phase occurring over the first 25 days of incubation, a “late” phase lasting from day 25 until sulfate was entirely consumed around day 50, and a “stationary” phase where only minor changes were observed in the chemical composition of the bioreactors. Visually, the initial light tan color of the reactors changed over the course of the experiment to a dark gray color coincident with the consumption of acetate and sulfate and the production of Fe^II^.

In the early phase, Fe^II^ began accumulating almost immediately in both AQDS+ and AQDS− bioreactors, with a significant step-wise increase at day 3 in the AQDS+ bioreactor (Fig 1). After one week, nearly 2 mM of Fe^II^ had accumulated in AQDS+ bioreactors compared to ~0.5 mM in AQDS− systems, consistent with the higher rate of Fe^II^ production in the AQDS+ bioreactors (Table 1). The production of Fe^II^ was concomitant with the consumption of acetate. The depletion of sulfate began almost as soon as the production of Fe^II^ in both the AQDS+ and AQDS− bioreactors, with no significant difference in sulfate consumption rates observed between the two sets of reactors. During the early phase, the concentration of sulfate decreased from 5 mM to ~3.5 mM while the concentration of acetate decreased from 10 mM to ~8 mM (Fig 1).

**Table 1:** Rates of Fe^II^ production and sulfate and acetate consumption.

		Fe^II^ production	Sulfate consumption	Acetate consumption
		rate	rate	rate
System	Phase^a^	mM day^-1^	mM day^-1^	mM day^-1^
Without AQDS (AQDS-)	Early	0.056 ± 0.001^b^	-0.067 ± 0.006	-0.081 ± 0.008
	Late	0.308 ± 0.019	-0.196 ± 0.016	-0.203 ± 0.009
With AQDS (AQDS+)	Early	0.549 ± 0.147	-0.061 ± 0.005	-0.081 ± 0.004
	Late	0.228 ± 0.017	-0.203 ± 0.021	-0.224 ± 0.013

^a^Early and late phases as indicated in Fig 1.

^b^Rates (average ± standard error) were calculated by least-squares regression of data during the linear portion of the indicated phase.

The late phase was demarcated by a substantial increase in the rates of consumption of sulfate and acetate in both AQDS+ and AQDS− bioreactors, coincident with a significant increase in the rate of Fe^II^ production in the AQDS- bioreactors (Fig 1 and Table 1) and continued Fe^II^ production in the AQDS+ bioreactors (albeit at a slower rate than during the early phase). Over this phase of the experiment, the amount of Fe^II^ more than doubled from 3.0 mM to 8.3 mM in the AQDS+ experiments and increased from 1.6 to 7.3 in the AQDS− bioreactors. No significant difference in the rate of sulfate consumption was observed between AQDS+ and AQDS− reactors during this phase; given that sulfate (unlike Fe^III^ oxides) is a soluble electron acceptor, it is perhaps not surprising that the presence of the electron shuttle AQDS did not increase the rate of sulfate reduction. The consumption of acetate ceased and the production of Fe^II^ diminished when all the sulfate was consumed, leading to a quiescent stationary phase over the final 100 days of the experiment during which only marginal geochemical changes were observed. Approximately 3.5 mM of acetate remained in both the AQDS+ and AQDS− reactors. At the final measurement, the amount of Fe^II^ present in the AQDS+ bioreactors was 8.3±0.4 mM compared to 7.3±0.5 mM in the AQDS–bioreactors, with 3.4±0.1 mM of acetate remaining the AQDS+ bioreactors and 3.0±0.1 mM in the AQDS–bioreactors. Although Fe^III^ and sulfate reduction reactions consume protons (i.e., raise the pH), the pH of bioreactors remained well-buffered over the duration of the incubation, only increasing from 7.5 to ~7.8. No changes in Fe^II^, acetate, and sulfate concentrations were observed in the sterile controls (S1 Fig in Supporting Information (S1 File)).

### XAFS spectroscopy

**Fig 2:**
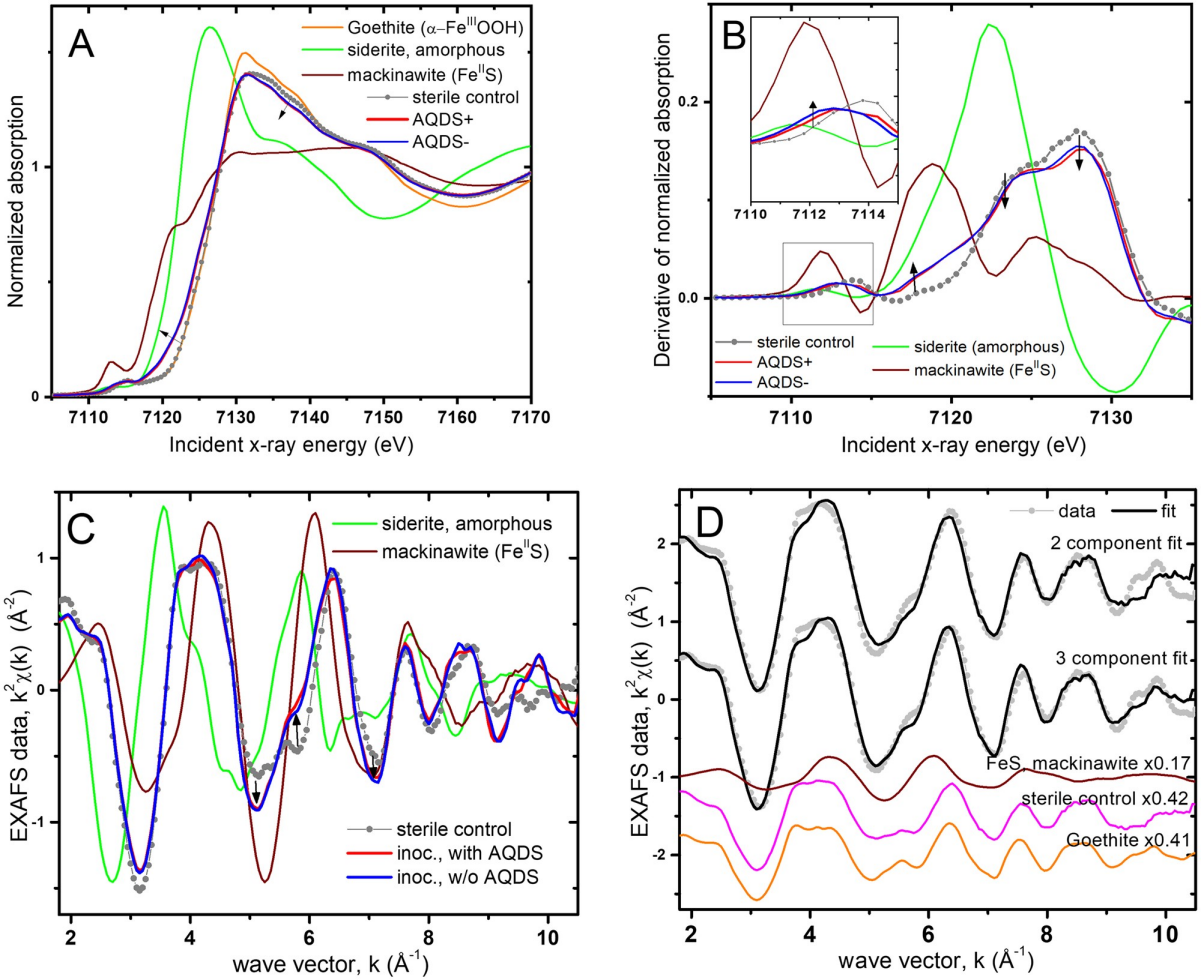
Fe XAFS analysis of bioreactor solids. Comparisons of XANES data from the bioreactors (with (AQDS+) and without AQDS (AQDS–)) to the sterile control and Fe^II^/Fe^III^ standards. The lines corresponding to the AQDS+ and AQDS- bioreactors (red and blue) nearly overlap. A) XANES data. Arrows indicate the spectral shifts from Fe^III^ to Fe^II^ standards. B) Derivative of the spectra in A. Arrows point in the direction of the spectral shifts towards the Fe^II^S standard. Inset shows detail in the pre-edge region (box). C) EXAFS data. Arrows point in the direction of spectral shifts towards the Fe^II^S standard. D) LC fit of the data from the inoculated AQDS–bioreactors using two and three endmembers (the data for AQDS+ and AQDS− bioreactors are the same within measurement uncertainty). The weighted components in the best fit are offset for clarity. Mackinawite and the sterile control were the best fit components in both 2- and 3-component LC models. The fit range is 2-10 Å^−1^ and the refined proportions are summarized in S1 Table in S1 File.

The Fe transformations in the bioreactors were characterized in subsamples taken after 144 days of incubation. Fig 2A and 2B compare the XANES spectra of the bioreactors to the sterile control and to Fe^II^/Fe^III^ standards. The sterile control shows the same edge position as the goethite standard, confirming that no Fe reduction occurred without inoculation. The edge position of the bioreactors samples is shifted in the direction of the Fe^II^ standard, which indicates partial reduction. Linear combination (LC) fits quantify the Fe^II^ content in the bioreators as 17% (±5%) of total solid-phase Fe (i.e., 5.2 mM Fe^II^), which is significantly lower than the Fe^II^ concentrations measured in the acid extracts (8.3±0.4 mM in the AQDS+ bioreactors and 7.3±0.5 mM in the AQDS–bioreactors). Since both microcoms contained only 0.9 mM dissolved Fe^II^, thee majority of the discrepancy between the Fe^II^ contents determined by XAFS and colorimetrically is likely due to an artifact of the acid extraction method [74] which can lead to an overestimation of Fe^II^ in systems containing sulfide and labile Fe^III^ oxides. The shape of the XANES features suggests FeS formation (Fig 2B), corroborated below by LC analysis of the EXAFS spectrum; additional details of the XANES analysis are presented in the SI.

The EXAFS spectra of the bioreactor solids are compared to the sterile control and to standards in Fig 2C. The AQDS+ and AQDS− bioreactors have identical EXAFS spectra, indicating that AQDS did not influence the final distribution of secondary mineralization products (SMPs). The proportions of SMPs in the bioreactors were quantified by LC fits of the EXAFS data. Fits with 2- and 3-components showed that the spectral combination of the sterile control and FeS provided a significantly better fit to the data than with any of the other Fe^II^-containing standards (Fig 2D, details in S1 File). The slight improvement of the fit with an additional goethite component suggests that the more labile oxides in the natural sienna starting phase are likely transformed first into reduced Fe^II^, leaving an increased relative proportion of goethite in the Fe^III^ pool. Overall, the x-ray spectroscopy results indicate the formation of FeS in the inoculated reactors and quantify its proportion as approximately 18% of solid-phase Fe. Minor SMPs (<10% of solid-phase Fe) could not be resolved by the LC analysis.

### Microbial community dynamics

**Fig 3:**
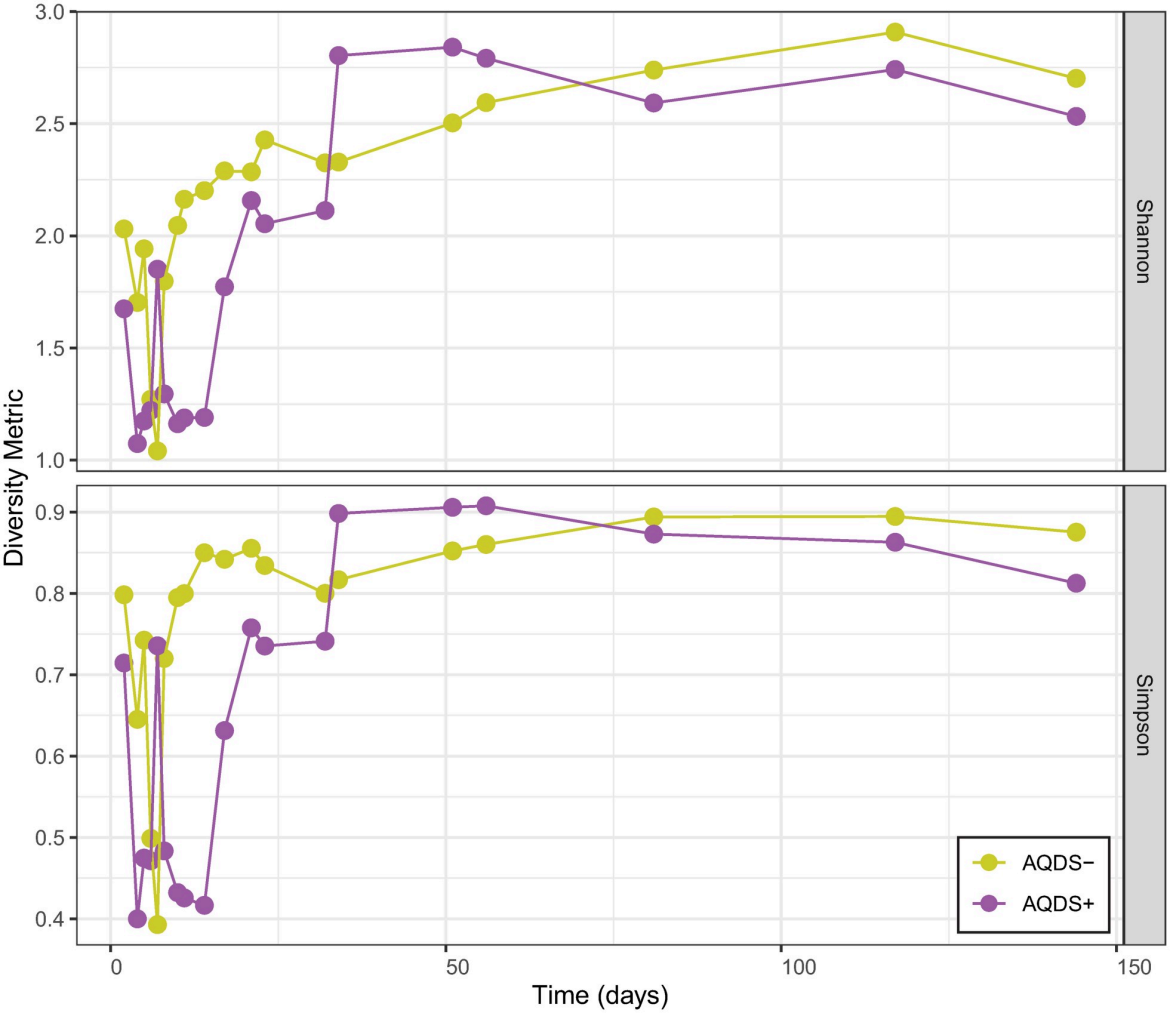
Changes in alpha diversity over time. Change over time in average alpha diversity as measured by the Shannon (top) and Simpson (bottom) indices during early and late phases of microbial community development in batch systems with (AQDS+) or without (AQDS–) added electron shuttle.

The distinct “early” and “late” phases observed in the geochemical composition of the bioreactors were mirrored by changes in the composition of the microbial community. A corresponding shift over time in the microbial communities was observed in both the alpha and the beta diversity of the system. The alpha diversity, a measurement of the diversity of distinct microbial OTUs within a particular sample, here measured by both Shannon’s and Simpson’s indices of diversity, showed an initial sharp decrease followed by an overall increase (Fig 3). The alpha diversity decreased more rapidly in the AQDS+ bioreactors than in the AQDS− system, where the decline in diversity reached its nadir approximately one week after the AQDS+ system.

**Fig 4:**
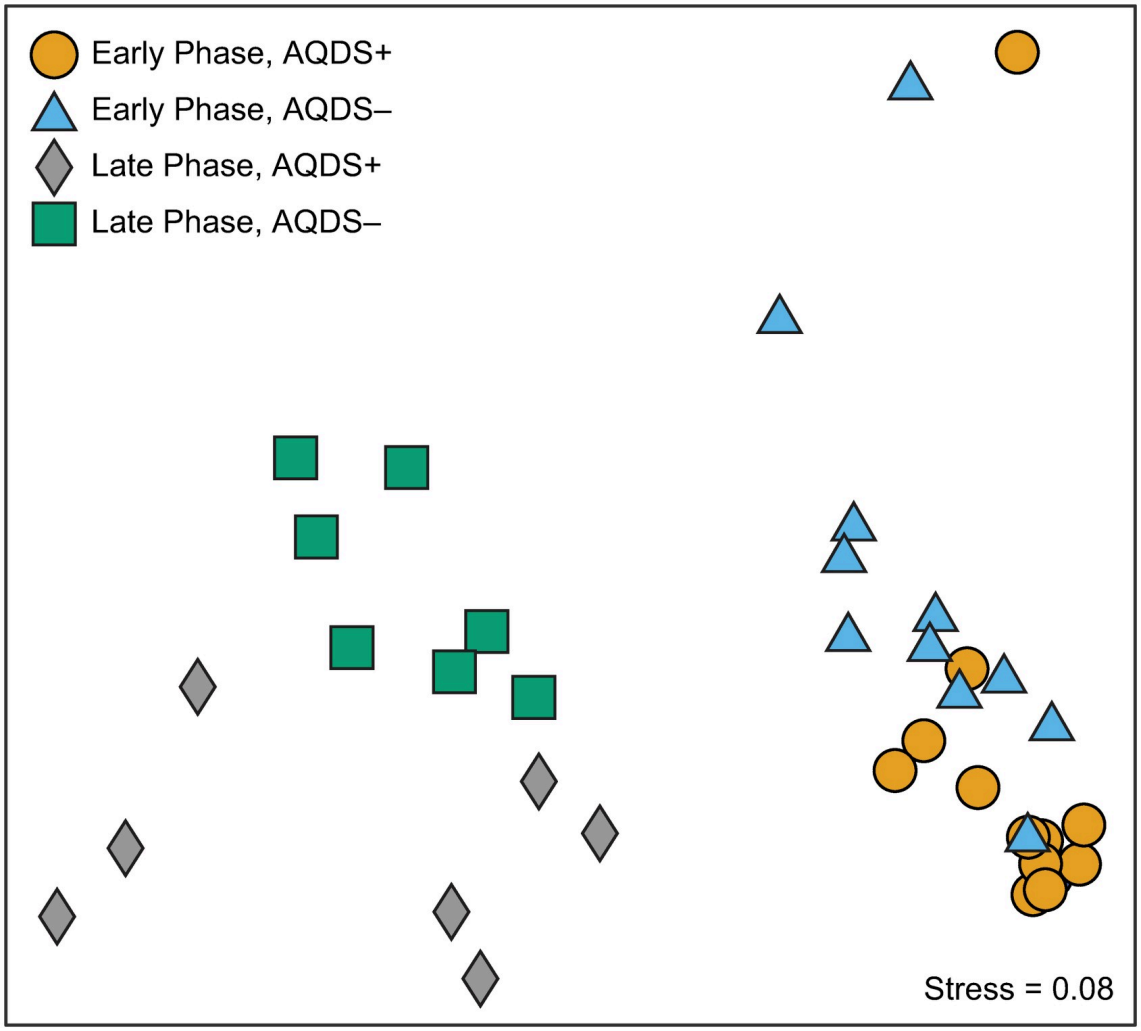
Nonmetric multidimensional scaling (NMDS) of microbial communities. Nonmetric multidimensional scaling (NMDS) of microbial communities in Fe^III^-, sulfate-, and acetate-amended bioreactors over time according to the presence (AQDS+) or absence (AQDS–) of the electron shuttle AQDS.

This distinction between early- and late-phase composition of the microbial community can also be seen in the NMDS plot of microbial communities from the bioreactors (Fig 4), where communities sampled at early time points cluster separately from those in the later phases. Based on the analysis of similarity metric (ANOSIM), the separation of early- from late-phase communities is statistically significant with an R_ANOSIM_ = 0.684 and a corresponding *p* < 0.001%. A value of R_ANOSIM_ > 0.75 indicates that two groups of microbial communities are almost entirely distinct from one another [75]. Lower values ranging from 0.75 to 0.25 indicate the groups overlap to some degree, while values < 0.25 indicate very little difference in the average composition of the two groups being compared. In the early phase, the AQDS+ and AQDS–communities do not differ significantly with an R_ANOSIM_ value of only 0.102 (*p* = 2.9%). Differences between the two treatments are much more distinct in the late phase, where R_ANOSIM_ = 0.505 (*p* = 0.060%).

**Fig 5:**
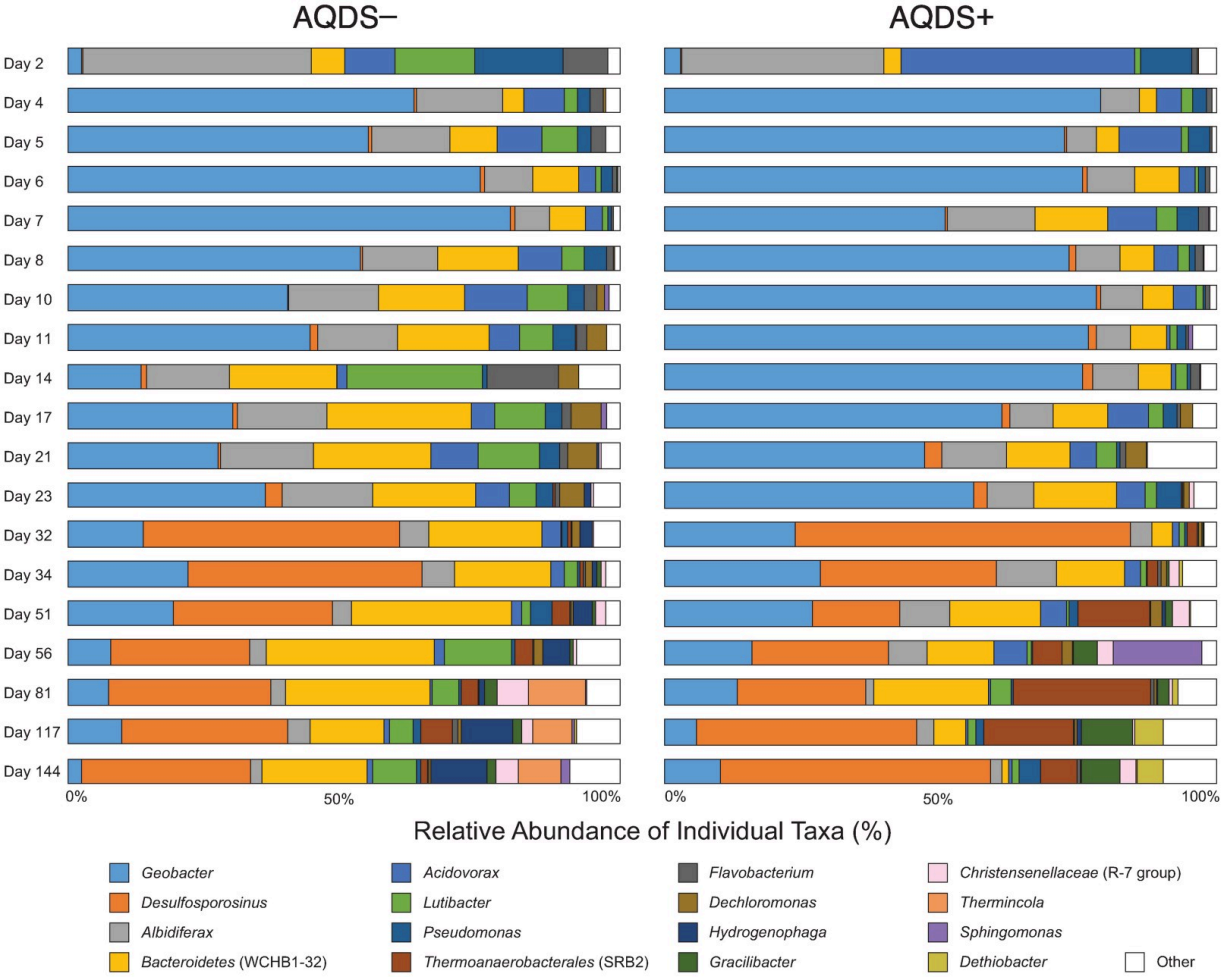
Changes in the relative abundance of microbial taxa over time. Comparison of the relative abundance of individual taxa detected by 16S rRNA gene amplicon sequencing in bioreactors amended with natural goethite, sulfate, and acetate in the presence (AQDS+) or absence (AQDS−) of the electron shuttle AQDS.

The steep decline in alpha diversity seen in Fig 3 corresponds with the onset of Fe^II^ production and the substantial increase between day 2 and day 4 of sequences belonging to the genus *Geobacter* (Fig 5). All currently known *Geobacter* isolates are capable of reducing Fe^III^ minerals [2]. Sequences classified as *Geobacter* accounted for only 2.5–3.0% of the total in both AQDS+ and AQDS–bioreactors on day 2, but that proportion increased to 79.0% by day 4 in AQDS+ bioreactors and 62.6% in AQDS–bioreactors. For the rest of the early phase of Fe^II^ production, *Geobacter* was the dominant taxonomic group, comprising 67.9% (AQDS+) and 46.7% (AQDS–) of the total taxonomic diversity, on average, prior to day 25. However, this dominance was more pronounced in the early phase of the AQDS+ bioreactors, where *Geobacter* sequences accounted for 56.1% of the total community compared to only 35.7% in AQDS–bioreactors. In addition to *Geobacter*, sequences most closely related to the dissimilatory metal-reducer *Albidiferax* were also abundant in the early phase. At day 2, *Albidiferax* sequences accounted for 36.6% of the total in AQDS+ bioreactors and 41.4% in AQDS–bioreactors. Only two days later, however, their share of the total had declined to 7.0% and 15.7%. *Albidiferax* sequences averaged only 16.2% and 10.9%, respectively, of the total relative abundance for the remainder of the early phase. *Albidiferax ferrireducens* (formerly *Rhodoferax ferrireducens*) is a member of the *Comamonadaceae* family that has been previously shown to reduce both Fe^III^ and Mn^IV^ but, interestingly, not AQDS [76].

In the late phase of the bioreactor experiments, the composition of the microbial community in both AQDS+ and AQDS–bioreactors shifted to one dominated by organisms most closely related to known sulfate-reducing bacteria. This is primarily due to a sharp increase in the relative abundance of sequences classified as *Desulfosporosinus* (Fig 5), a genus of known sulfate-reducing bacteria that have been observed to proliferate in organic C-amended soils and sediments from a variety of environments [56,77–82]. Sequences classified as this genus accounted for only 0.8–1.1% of all sequences in the early phase of the experiment, but their relative abundance increased sharply from 2.3% to 60.5% of all sequences in the AQDS+ experiments and from 3.1 to 46.4% in AQDS–between day 23 and day 32 of the experiments. *Desulfosporosinus* sequences remained the most abundant taxa in the late phase stage of the experiment, with an average relative abundance of 35.0% (AQDS+) and 33.2% (AQDS–) through day 144. While *Desulfosporosinus* sequences were the most abundant for taxa related to known sulfate reducers, sequences classified within the phylotype “SRB2” within the class *Thermoanaerobacterales* were present at an abundance ranging from 5.3–24.9% after day 51. While the phylogenetic placement of taxa within this class remains uncertain, some taxa within the group are associated with reductive S-cycling [83–85].

**Fig 6:**
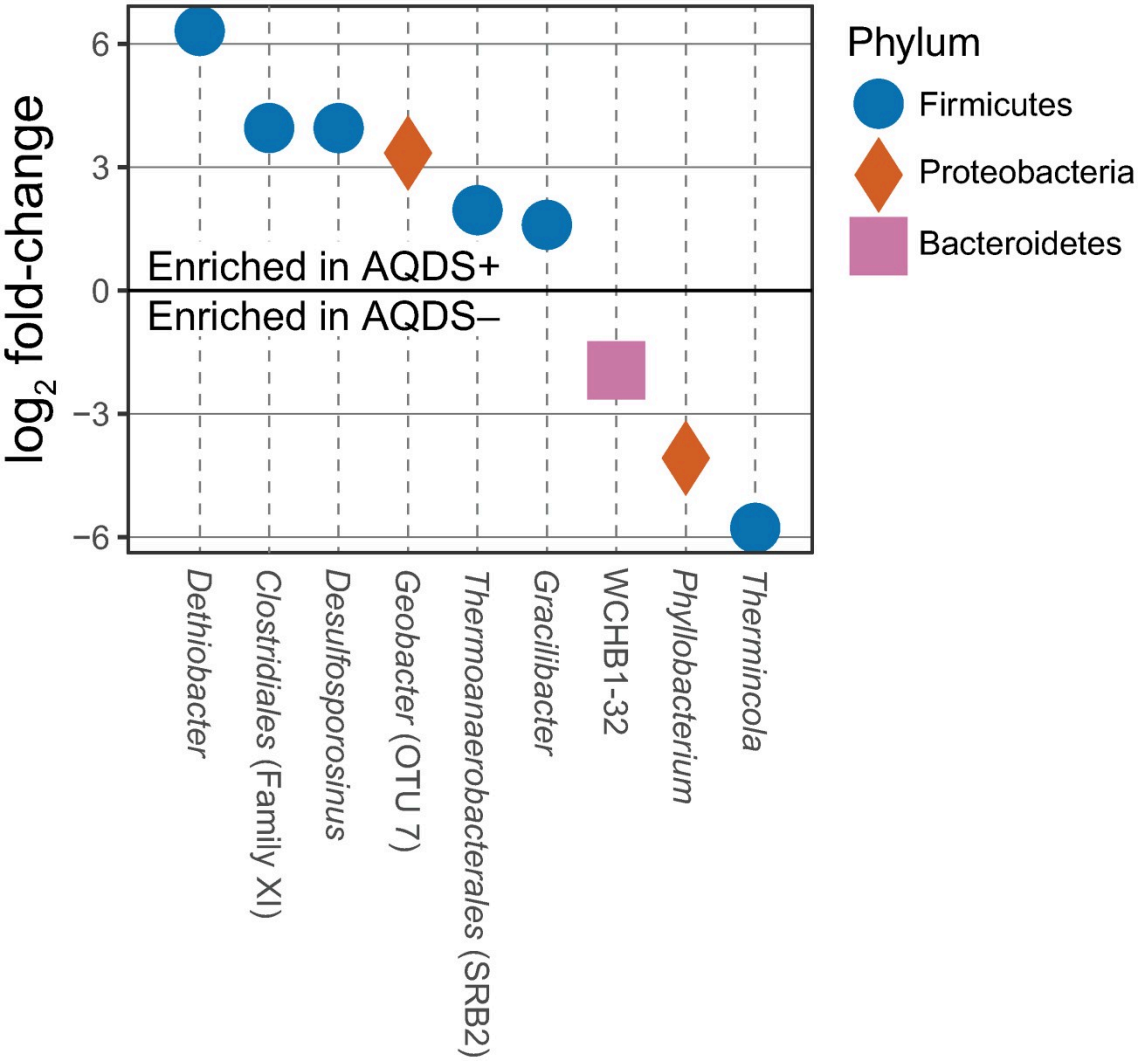
Comparisons of OTUs significantly more or less abundant between the late-phase AQDS+ and AQDS–bioreactors. Differentially-abundant OTUs in AQDS+ and AQDS–bioreactors as determined by edgeR. OTUs with a positive log2-fold change value are more relatively abundant in AQDS+ bioreactors, while those with a negative value are more relatively abundant in AQDS–bioreactors.

**Fig 7:**
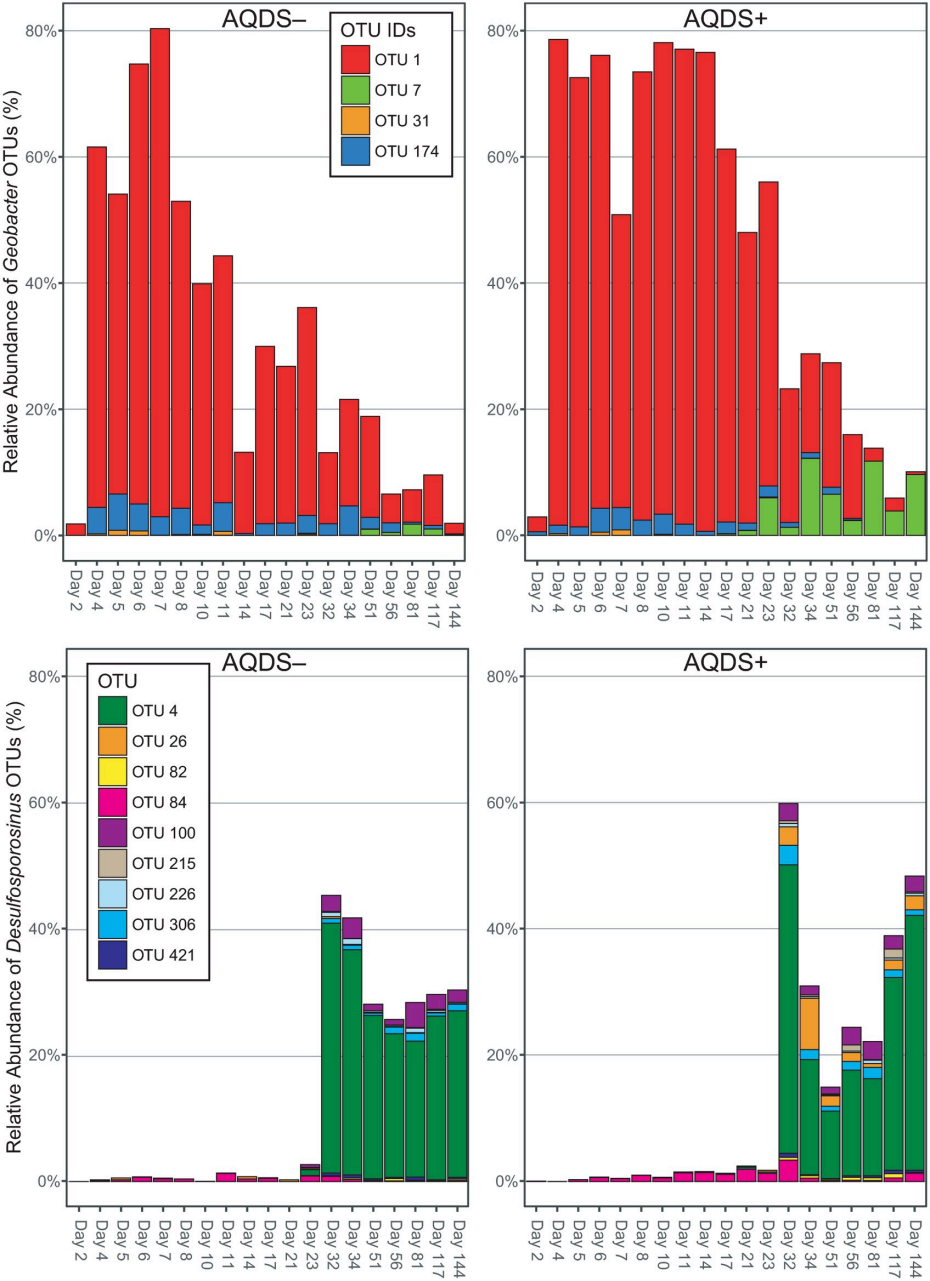
Relative abundance of *Geobacter* and *Desulfosporosinus*. Relative abundance of operational taxonomic units (OTUs) classified as *Geobacter* or *Desulfosporosinus* in AQDS+ and AQDS–bioreactors.

Comparisons of differentially abundant OTUs made using edgeR identified 9 OTUs that were significantly more or less abundant between the late-phase AQDS+ and AQDS–bioreactors (Fig 6). An OTU classified as genus *Dethiobacter*, a member of phylum *Clostridia* known to utilize thiosulfate, elemental sulfur and polysulfide as electron acceptors (but not sulfate), was considerably more abundant in the late-phase AQDS+ bioreactors compared to AQDS–. Conversely, the most differentially abundant OTU in the AQDS–bioreactors was most closely related to *Thermincola*, a Fe^III^-reducing member of phylum *Clostridia*. Other differentially abundant OTUs include *Gracilibacter*, *Phyllobacterium*, and the SRB2 family of order *Thermoanaerobacterales*. OTUs classified as *Geobacter* and *Desulfosporosinus* were also more abundant in the AQDS+ bioreactors, although these particular OTUs were not the dominant OTUs responsible for most of the relative abundance of these particular taxa. The most differentially abundant *Geobacter* OTU is OTU-7, which was more than 10 times as abundant in the late-phase of AQDS+ bioreactors than it was in those without AQDS. This particular OTU was barely detectable in the early, *Geobacter*-dominated phase of the AQDS+ treatment but grew to become the dominant *Geobacter* OTU over the late, *Desulfosporosinus*-dominated phase (Fig 7).

## Discussion

The addition of the electron shuttle AQDS did increase the initial rate of Fe^III^ reduction (Fig 1 and Table 1); however, it had little effect on the composition of the microbial community during this phase (Fig 5). The increase in Fe^II^ in both AQDS+ and AQDS–bioreactors corresponded directly to an increase in the relative abundance of sequences classified as *Albidiferax* and *Geobacter*. A single OTU dominated the *Geobacter* in the AQDS–bioreactors throughout the experiment and in the AQDS+ bioreactors through day 56 (Fig 7). Both *Albidiferax* and *Geobacter* are known to reduce ferric minerals like the goethite present in the natural sienna amendment. *Geobacter* in particular has been shown to predominate in sedimentary environments where Fe^III^ reduction is occurring [86–91], typically in response to acetate biostimulation [56,92–97]. This pattern is well-established, as *Geobacter* blooms have been observed in Fe^III^- and acetate-amended bioreactors using material from rice paddies [98], marshes [99], and aquifer sediment [100,101]. *Geobacter* has also been enriched in acetate-amended experimental systems where AQDS (not Fe^III^) was added as the sole electron acceptor [102]. Some other taxa (e.g., *Acidovorax*) were abundant at day 2, but these were quickly overtaken in dominance by *Geobacter*.

Given the diversity of organisms capable of reducing synthetic (e.g., AQDS) and naturally occurring quinones (e.g., humic substances) [44], we had hypothesized that the presence of AQDS would lead to greater microbial diversity in the AQDS+ bioreactors during Fe^III^ reduction due to recruitment of non-metal-reducing, quinone-respiring organisms. However, this was not the case under our experimental conditions. In both AQDS+ and AQDS–bioreactors diversity declined at the onset of Fe^II^ production, with sequences classified as *Geobacter* becoming more abundant. Indeed, *Geobacter* were even more dominant in the AQDS+ systems than in AQDS–; which in retrospect is perhaps not unexpected given their ability to use both Fe^III^ and AQDS as terminal electron acceptors for anaerobic respiration [2]. A similar enhancement in *Geobacter* abundance was reported by Rowland et al. [100] in microcosm studies where sediments were amended with acetate alone or acetate and AQDS (50 μM; an order of magnitude lower AQDS concentration than in our study) and by Chen et al [103] where the relative abundance of *Geobacter* in rice paddy soil incubations increased with increasing AQDS concentration. It is possible that AQDS may be toxic to some members of the Fe^III^-reducing community, potentially decreasing diversity. Direct evidence of the potential toxicity of AQDS to bacteria is lacking; however, circumstantial evidence suggests that AQDS toxicity is not likely to be an issue in our systems containing such low (100 μM) concentrations of the quinone molecule. In acetate-amended sediment incubations, lower Fe^II^ concentrations were observed in systems containing 250 μM AQDS compared with 50 μM, the next lowest concentration examined [21]. However, AQDS concentrations as high as 20 mM showed no inhibition of Fe^II^ production relative to concentrations as low as 10 μM in rice paddy soil incubations [103]. Furthermore, pure culture studies with *Geobacter sulfurreducens* and *Shewanella putrefaciens* CN32 showed no inhibition of Fe^II^ production with 500 μM and 1000 μM AQDS, respectively [20,104].

Similar to the trajectory observed following acetate injection in a uranium-contaminated aquifer in Rifle, Colorado [56] (the same location from which the sediment used to inoculate the bioreactors was obtained), the initial bloom of *Geobacter* was followed by a period of sulfate reduction and the increased relative abundance of sequences associated with sulfate-reducing bacteria. At both Rifle and in our experiment, the most abundant taxa associated with sulfate reduction were of the genus *Desulfosporosinus*. Initially at less than <1% of the total community in these systems, *Desulfosporosinus* sequences eventually came to dominate the microbial community during the latter stages of the experiment, where they accounted for roughly one-third of all sequences in both the AQDS+ and AQDS–bioreactors (Fig 5). As with the *Geobacter* OTUs, a single *Desulfosporosinus* OTU dominated in both AQDS+ and AQDS–bioreactors (Fig 7). In addition to the bioreactors in this study, as well as an earlier study from our group [105], *Desulfosporosinus* have previously been found in acetate-amended sediments [78,106,107].

The preponderance of *Desulfosporosinus* in these acetate-amended systems is perhaps unexpected given the inability of all previously cultivated *Desulfosporosinus* spp. to couple acetate oxidation to dissimilatory sulfate reduction [108–118]. However, the increase in the relative abundance of *Desulfosporosinus* in our bioreactors was coincident with a sharp increase in the rate of sulfate consumption (Fig 1 and Table 1), suggesting that they are active contributors to sulfate reduction in this system. *G*. *sulfurreducens* can oxidize acetate by syntrophic association with hydrogen-oxidizing anaerobic partners [119,120] and many *Desulfosporosinus* spp. can use H_2_ as an electron donor for dissimilatory sulfate reduction [109,110,114,116,118,121] suggesting the possibility for sulfate reduction via a syntrophic association between *Geobacter* and *Desulfosporosinus*.

Of particular note is that the majority of Fe^II^ production actually occurred during the “sulfate-reducing” latter phase of the experiment. During the early phase of the experiment dominated by *Albidiferax* and *Geobacter*, <10% of the total amount of Fe^III^ added was reduced. Indeed, 64% and 78% of the total amount of Fe^II^ produced occurred during the “sulfate-reducing” phase in AQDS+ and AQDS–bioreactors, respectively, likely driven by the reduction of Fe^III^ by the sulfide produced by *Desulfosporosinus* and other SRB [122]. These results are consistent with recent findings highlighting the potential importance of sulfur-driven reactions in the biogeochemical cycling of iron in sedimentary environments. Because iron reduction is strongly pH-dependent, experimental and modeling evidence suggests that under sulfidic, alkaline conditions, Fe^III^ reduction by metal-reducing bacteria likely proceeds primarily via an electron shuttling pathway mediated by S^0^ [31]. Even under circumneutral conditions, laboratory and field studies suggest that sulfur cycling can play a significant role in Fe^III^ reduction in freshwater and marine environments [122–125].

These results call into question the paradigm of parsing out geomicrobiological reactions into “iron-reducing” and “sulfate-reducing” phases. This traditional conception of terminal electron accepting processes in the subsurface, while long-established (e.g., [126]), has increasingly been called into question by both theoretical and experimental observations in both the field and laboratory. Iron reduction and sulfate reduction have frequently been observed to co-occur in sedimentary environments [86,87,127–129], and modeling results predict that the co-occurrence of these processes may even benefit both groups [130,131]. In our results, sulfate reduction began essentially consequent with iron reduction. While some of the sulfate may have been consumed by assimilatory sulfate reduction during the growth of other organisms, this is unlikely as there was no difference in the rate of sulfate consumption between AQDS+ and AQDS–systems. If the growth of iron reducers was responsible for the decrease in the concentration of sulfate, the rate of sulfate consumption would have been greater during the early phase in AQDS+ bioreactors. While initially higher rates of growth, particularly in the presence of electron shuttles, may give iron reducers an early advantage under growth-stimulating conditions, such dynamics may ultimately bear little resemblance to the processes that occur in most aquifers.

## Supporting information

S1 File Supporting information on geochemical conditions in the sterile controls and Fe mineralogy and XAFS analysis. Fe^II^, acetate, and sulfate concentrations over time in the sterile controls as well as a detailed description of the characterization of Fe mineralogy in natural sienna and details of the XAFS analysis of bioreactor solids.
(DOCX)
