# Supplementary material for: Biogeochemical dynamics and microbial community development under sulfate- and iron-reducing conditions based on electron shuttle amendment
Source: PLoS One. 2021 May 20;16(5):e0251883. doi: 10.1371/journal.pone.0251883 (PMC8136678; doi:10.1371/journal.pone.0251883)
Supplement: S1 File — FeII, acetate, and sulfate concentrations over time in the sterile controls as well as a detailed description of the characterization of Fe mineralogy in natural sienna and details of the XAFS analysis of bioreactor solids. (DOCX) [file pone.0251883.s001.docx]

**Supporting Information**

**Biogeochemical dynamics and microbial community development under sulfate- and iron-reducing conditions based on electron shuttle amendment**

**Theodore M. Flynn^1^****^†^, Dionysios A. Antonopoulos^1^, Kelly A. Skinner^1^, Jennifer M. Brulc^1^, Eric Johnston^1^, Maxim I. Boyanov^1,2^, Man Jae Kwon^1,3^, Kenneth M. Kemner^1^, and Edward J. O’Loughlin^1^***

^1^Biosciences Division, Argonne National Laboratory, Lemont, IL, 60439

^2^Bulgarian Academy of Sciences, Institute of Chemical Engineering, Sofia, Bulgaria

^3^Department of Earth and Environmental Sciences, Korea University, Seoul, South Korea

*Corresponding author: Argonne National Laboratory, 9700 South Cass Ave., Lemont, IL, USA, 60439. Email: oloughlin@anl.gov.

^†^Present address: California Department of Water Resources, West Sacramento, CA 95691

**
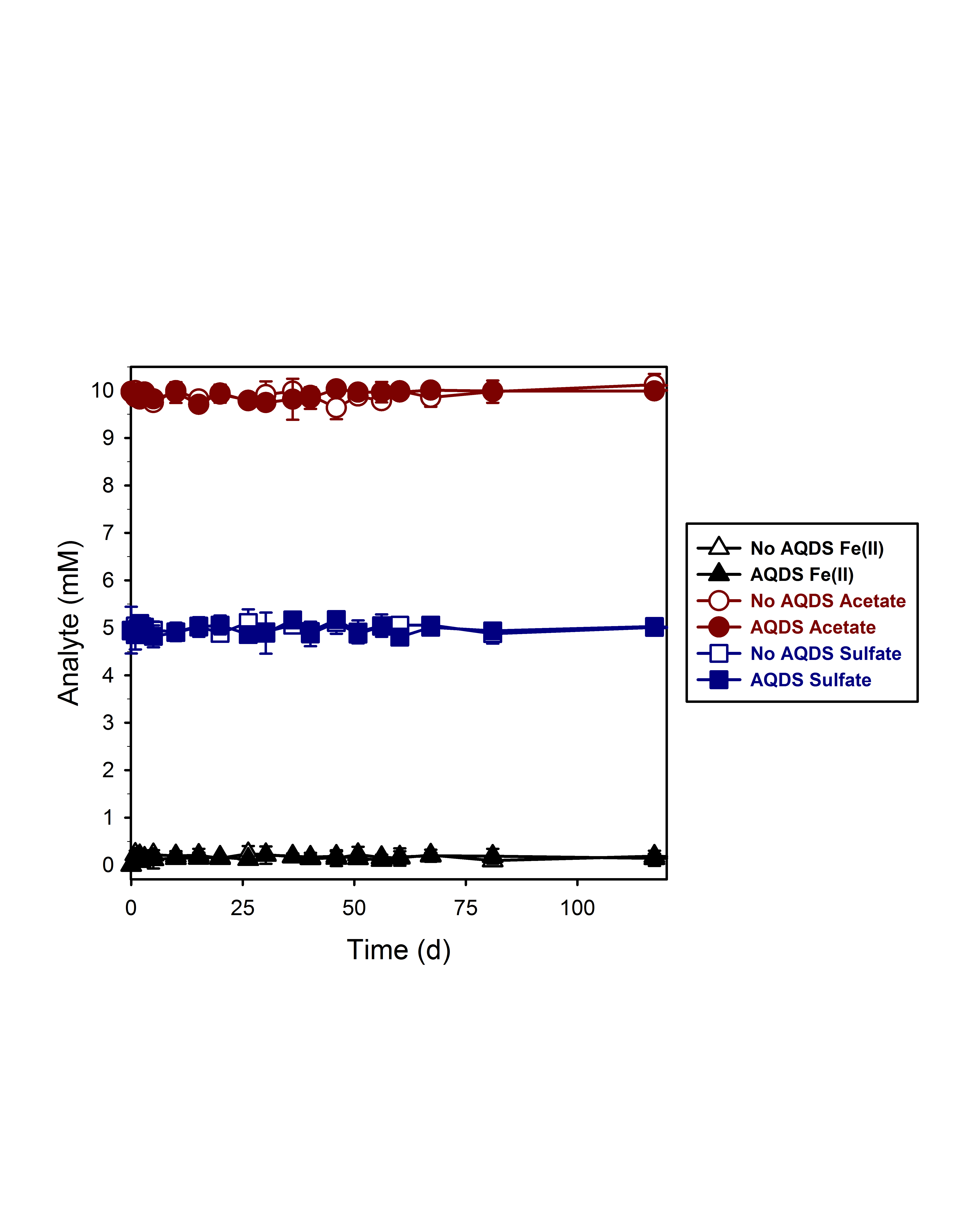
**

**S1 Fig**. Fe(II), acetate, and sulfate concentrations in the sterile controls.

**Characterization of Natural Sienna.** Iron(III) was provided as natural sienna, an iron-rich earth mined from the Ochre deposits in the Provence region of France (The Earth Pigments Co.). The mineralogy and Fe speciation in natural sienna were determined by powder X-ray diffraction (pXRD) and X-ray absorption near edge structure (EXAFS) spectroscopy. The sample for pXRD analysis was prepared by packing natural sienna as received < 50 µm powder) into the sample holder followed by analysis with a Rigaku MiniFlex X ray diffractometer with Ni-filtered Cu Kα radiation, scanned between 5° and 80° 2θ at a speed of 0.1° 2θ min^-1^. The XRD pattern was analyzed with the JADE 9 software package (MDI, Livermore, CA) to remove the background through polynomial fitting as well as the Kα2 component. For EXAFS analysis, a stock suspension of the natural sienna material was filtered through a 0.22 μm filter and the X-ray absorption spectrum of the solids deposited on the membrane was measured in transmission mode (XAS analysis procedures are detailed in the next section).

The pXRD data for natural sienna indicate that the crystalline phases in this material are primarily quartz and goethite (α-FeOOH), with indications of other unidentified peaks (**S2 Fig**). The identification of goethite as the predominant Fe phase in natural sienna is corroborated by the EXAFS analysis. The spectrum and its Fourier transform closely resemble that of the crystalline goethite standard (**S3A Fig**). The slight amplitude suppression relative to the pure crystalline standard may indicate that goethite is present as nm-sized particles or that other Fe-containing phases are also present. Linear combination (LC) fits with a set of Fe(III) standards (listed in the next section) resulted in a best fit with 85% goethite and 15% lepidocrocite; however, fits with other iron oxides (e.g., ferrihydrite) for the ~15% component were statistically equivalent and could not be excluded. Overall, the LC EXAFS analysis indicates that at least 85% of the Fe in the natural sienna amendment is in the form of goethite.


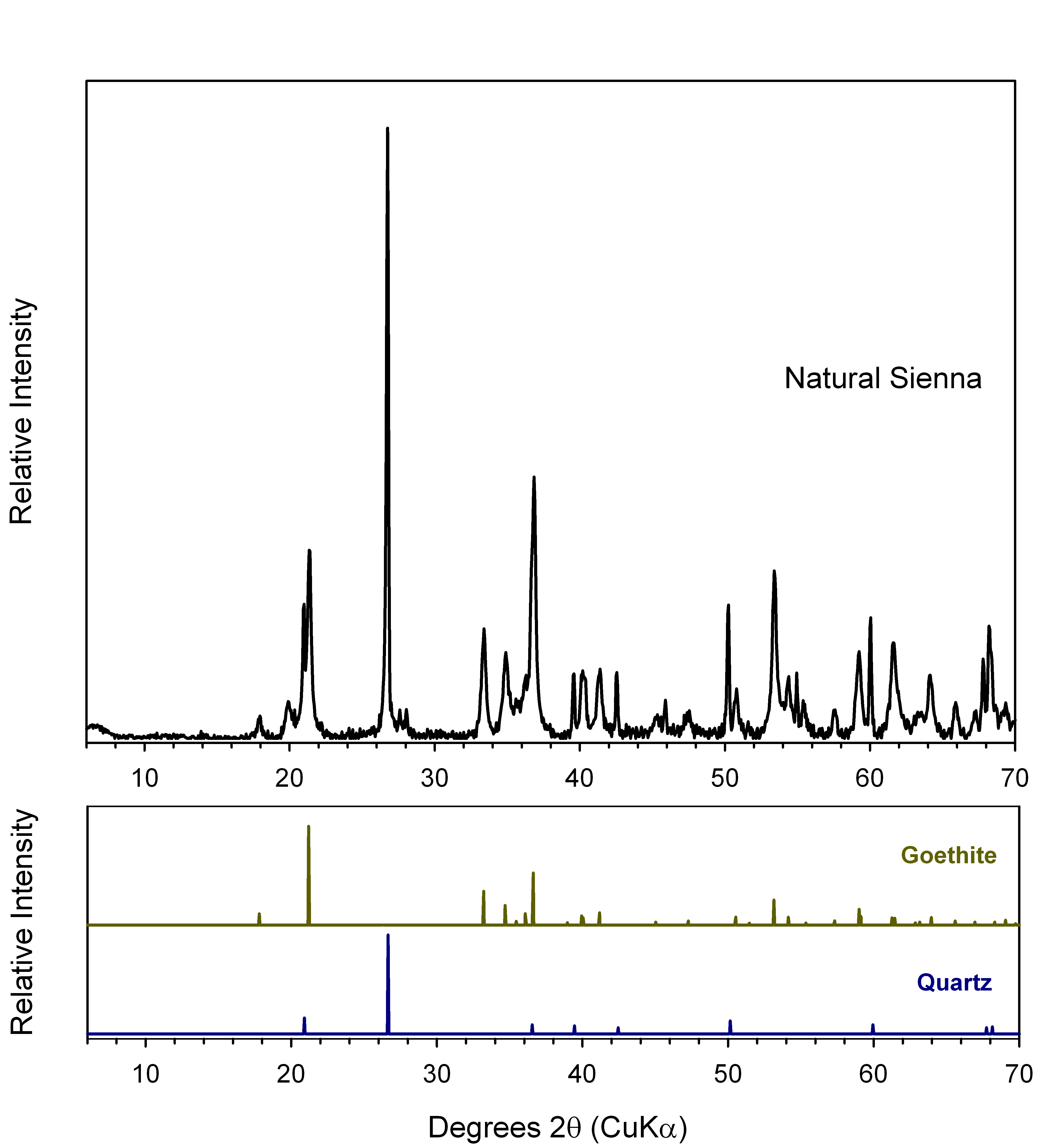


**S2 Fig**. pXRD pattern of natural sienna compared with reference patterns of goethite and quartz.


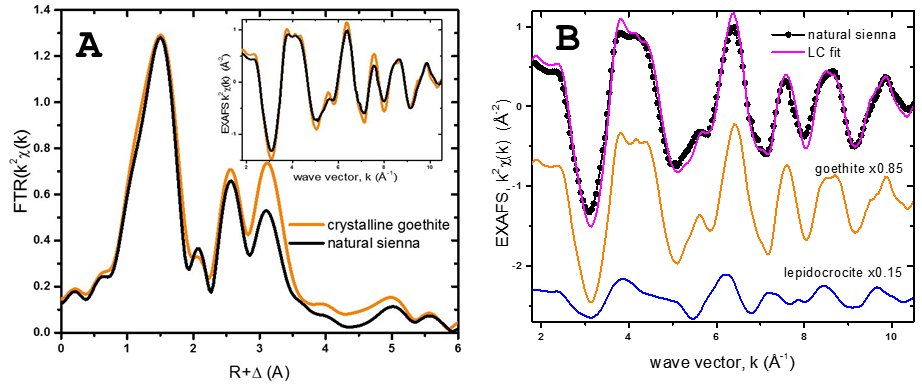
 **S3 Fig**. (A) Fe K-edge EXAFS data and Fourier transform for the natural sienna sample and the goethite standard. (B) Linear combination fit with Fe(III) oxide standards. The weighted components in the fit are offset vertically for clarity and the refined weights are noted.

**X-ray absorption spectroscopy analysis of bioreactor solids.**

Analysis of the spectra included comparisons to Fe-containing standards followed by linear combination (LC) fits to extract the spectral weights of up to three standards that best describe the bioreactor spectra. Combinatorial LC fitting involves refining all combinations of the spectra within a standards data set and ranking them according to a goodness-of-fit indicator (R-factor). Initial fits were carried out with 2 components and a third component was included only when the improvement in the fit was considered significant (i.e., the R-factor was decreased by more than 30% relative to the best fit with two components). The standards dataset used here included the spectrum from the sterilized control reactor and potential biotransformation products, such as ferrihydrite (Fh), lepidocrocite (Lp), goethite (Gt), hematite (Hem), magnetite (Mag), phosphate-stabilized carbonate green rust (GR), freshly-precipitated and annealed siderite (amSid and crystSid), vivianite (Viv), dissolved Fe(II) (Fe2aq), Fe(II) adsorbed to carboxyl resin (Fe2ads), Fe(OH)_2_, synthetic ferrous hydroxy-carbonate (FHC), mackinawite (FeS), ferrous oxide (FeO). The Fe standards were measured and characterized in previous work at the same beamline [1-6]. The polycrystalline Fe powders were mounted on the adhesive side of Kapton tape and their absorption spectra were measured in transmission. Solution samples were mounted in 1.5mm thick sample holders with Kapton film windows and the spectrum was measured in fluorescence. Adsorbed Fe standards were mounted as wet pastes in a 1.5mm thick sample holder with Kapton windows and measured in fluorescence mode. Normalization and background removal of the data was done using the program AUTOBK [7]. The combinatorial LC fits were performed using the program ATHENA [8].

**Fe valence in the bioreactor solids.** The XANES data from the bioreactor solids after 144 days of incubation are compared to spectra from the sterile control and from Fe^II^/Fe^III^ standards in **Fig 2A** (main text). The sterile control shows the same edge position and shape as the goethite standard, indicating that no reduction occurred without inoculation. The shift of the edge position to lower energy in the inoculated reactors (see arrows in the direction of the Fe^II^ standards) indicates that partial reduction occurred. The reactors with and without added AQDS show the same spectrum, suggesting that AQDS did not influence the final extent of reduction. Linear combination fits with Fe^II^ and Fe^III^ standards quantify the Fe^II^ content in the inoculated reators as 17% (±5%) of total solid-phase Fe (analysis not shown). The features in the derivative XANES spectra suggest the formation of sulfide-coordinated Fe^II^ (**Fig 2B**). To interpret the spectral differences between the bioreactors and the control, we note that the measured XAS is a molar ratio weighed linear combination of the spectra from the different Fe phases present in the solids. That is, if Fe reduction leads to the appearance of an oxygen-coordinated Fe^II^ phase such as the siderite standard shown, the spectrum would shift higher than the control near 7123-7126 eV and if an FeS solid forms the spectrum should shift lower than the control in the direction of the FeS standard. The presence of the downward shift near 7123-7126 eV therefore suggests the presence of FeS. Additional evidence for FeS formation is observed in the pre-edge features near 7112 eV, where the spectra diverge from the sterile control in the direction of the FeS standard and not in the direction of the siderite standard (arrow in inset of **Fig 2B**). The discussed trends are not conclusive and are presented here as qualitaive corroboration of the more rigorous LC fits of the EXAFS data in the next section.

**Fe speciation in the bioreactor solids.** **Fig 2C** in the main text compares the Fe K-edge EXAFS data obtained from the inoculated reactors to spectra from the sterile control and standards. Comparisons to the spectra of O-coordinated (green rust, adsorbed Fe^II^, siderite, vivianite) and S-coordinated (mackinawite, pyrite) Fe^II^ standards in our library showed that the shifts in the inoculated samples from the sterile control were in the direction of the mackinawite standard, suggesting the formation of sulfide-coordinated Fe^II^ in the bioreactors. The more prominent features (where the S-standards contribute in oposite directions relative to the O-standards and therefore allow disambiguation) are the downward shifts relative to the sterile control near 5.2 Å^-1^ and 7.1 Å^-1^ (arrows in **Fig 2C**). The combinatorial LC analysis confirms the qualitative trends. The potential SMPs tested included ferrihydrite, lepidocrocite, goethite, hematite, magnetite, phosphate-stabilized carbonate green rust, freshly-precipitated and annealed siderite (amSid and crystSid), vivianite, dissolved Fe(II), Fe(II) adsorbed to carboxyl-functionalized resin, Fe(OH)_2_, synthetic ferrous hydroxy-carbonate (FHC), mackinawite (FeS), and ferrous oxide (FeO). Weighted spectral combinations of the sterile control with up to 2 additional SMP standards were refined against the data from the bioreactors and the results are summarized in **Fig 2C** and **S1 Table**. The 2-component fits showed that the sterile control combined with FeS provided a significantly better fit to the data than any of the other reduced Fe standards, with the next best fit having about a 50% larger R-factor (not shown). The best fit with 3 components again included the sterile control and FeS, with only goethite providing a significant R-factor improvement over the two-component fit. The apparent need to include both goethite and the sterile control to reproduce the experimental data can be rationalized by the fact that goethite is the primary phase in natural sienna, so the linear combination of the sterile control and the goethite standard emulates a change in the proportions of goethite and the minor components in natural sienna which may have been preferentially reduced during these experiments. In summary, the LC analysis of the EXAFS data supports the formation of FeS in the bioreactors and quantifies its amount as approximately 18% of solid-phase Fe.

**TABLES**

Table S1. Refined proportions^a^ of the endmember components in the LC fits.

| Fit endmembers: | Sterilized control | FeS | Goethite | R-factor |
| --- | --- | --- | --- | --- |
| Inoculated bioreactor without AQDS | | | | |
| 2 component LC fit | 83% | 17% |  | 0.041 |
| 3 component LC fit | 42% | 17% | 41% | 0.018 |
| Inoculated bioreactor with AQDS | | | | |
| 2 component LC fit | 82% | 18% |  | 0.041 |
| 3 component LC fit | 42% | 19% | 39% | 0.019 |

a) Uncertainties from this analysis are estimated at ±5%.

**References**

1. Dong Y, Sanford RA, Boyanov MI, Kemner KM, Flynn TM, O'Loughlin EJ, et al. *Orenia metallireducens* sp. nov. strain Z6, a novel metal-reducing member of the phylum firmicutes from the deep subsurface. Appl Environ Microbiol. 2016;82(21):6440-53. Epub 2016/08/28. doi: 10.1128/AEM.02382-16. PubMed PMID: 27565620.

2. Kwon MJ, O'Loughlin EJ, Boyanov MI, Brulc JM, Johnston ER, Kemner KM, et al. Impact of organic carbon electron donors on microbial community development under iron- and sulfate-reducing conditions. PLoS ONE. 2016:1-22. doi: 10.1371/journal.pone.0146689.

3. Kwon MJ, Boyanov MI, Antonopoulos DA, Brulc JM, Johnston ER, Skinner KA, et al. Effects of dissimilatory sulfate reduction on FeIII (hydr)oxide reduction and microbial community development. Geochim Cosmochim Acta. 2014;129(12):4570-6. doi: 10.1016/j.gca.2013.09.037.

4. Kwon MJ, Yang J-S, Shim MJ, Boyanov MI, Kemner KM, O’Loughlin EJ. Acid extraction overestimates the total Fe(II) in the presence of iron (hydr)oxide and sulfide minerals. Environmental Science & Technology Letters. 2014;1(7):310-4. doi: 10.1021/ez500152h.

5. O'Loughlin EJ, Boyanov MI, Flynn TM, Gorski C, Hofmann SM, McCormick ML, et al. Effects of bound phosphate on the bioreduction of lepidocrocite (γ-FeOOH) and maghemite (γ-Fe_2_O_3_) and formation of secondary minerals. Environ Sci Technol. 2013;47(16):9157-66. Epub 2013/08/06. doi: 10.1021/es400627j. PubMed PMID: 23909690.

6. Boyanov MI, O'Loughlin EJ, Roden EE, Fein JB, Kemner KM. Adsorption of Fe(II) and U(VI) to carboxyl-functionalized microspheres: The influence of speciation on uranyl reduction studied by titration and XAFS. Geochim Cosmochim Acta. 2007;71(8):1898-912. doi: DOI 10.1016/j.gca.2007.01.025. PubMed PMID: ISI:000245860500002.

7. Newville M, Livinš P, Yacoby Y, Rehr JJ, Stern EA. Near-edge x-ray absorption fine structure of Pb: A comparison of theory and experiment. Phys Rev B. 1993;47(21):14126-31.

8. Ravel B, Newville M. ATHENA, ARTEMIS, HEPHAESTUS: Data analysis for X-ray absorption. Journal of Synchrotron Radiation. 2005;12(4):537-41. Epub 2005/06/22. doi: 10.1107/S0909049505012719. PubMed PMID: 15968136.
